# The MOHIP-14^PW^ (Modified Oral Health Impact Profile 14-Item Version for Pregnant Women): A Real-World Study of Its Psychometric Properties and Relationship with Patient-Reported Oral Health

**DOI:** 10.3390/healthcare10030461

**Published:** 2022-03-01

**Authors:** Chengwu Yang, Shulamite S. Huang, Tiffany A. Moore Simas, Hugh Silk, Judith A. Savageau, Stefanie L. Russell

**Affiliations:** 1 Department of Population and Quantitative Health Sciences, UMass Chan Medical School, Worcester, MA 01655, USA; tiffanya.mooresimas@umassmemorial.org; 2 Department of Obstetrics and Gynecology, UMass Chan Medical School, Worcester, MA 01655, USA; 3 Department of Epidemiology and Health Promotion, College of Dentistry, New York University, New York, NY 10010, USA; shulamite.huang@nyu.edu; 4 Department of Family Medicine and Community Health, UMass Chan Medical School, Worcester, MA 01655, USA; hugh.silk@umassmemorial.org (H.S.); judith.savageau@umassmed.edu (J.A.S.); 5 Department of Oral Surgery, Bellevue Hospital, New York, NY 10016, USA

**Keywords:** oral health related quality of life (OHQoL), real-world study (RWS), OHIP-14, pregnant women, psychometrics, factor analysis, confirmatory factor analysis (CFA), exploratory factor analysis (EFA)

## Abstract

*Background*: The 14-item version of the Oral Health Impact Profile (OHIP-14) has been widely used as a measure for oral health-related quality of life (OHQoL) since its publication in 1997. However, few studies have examined its psychometric properties and relationship with patient-reported oral health in pregnant women. *Aim*: To offer empirical evidence for appropriate use of the OHIP-14 among pregnant women in research and clinical practice. *Objectives*: (1) to empirically investigate the psychometric properties of the OHIP-14, (2) to modify it into the MOHIP-14^PW^ (modified OHIP-14 for pregnant women), and (3) to compare their relationships with patient-reported oral health in pregnant women. *Methods*: In this real-world study (RWS) from suburban New York clinics, we collected OHIP-14 data from 291 pregnant women and assessed its psychometric properties at the item-, dimension-, and measure-level, including confirmatory factor analysis (CFA) and exploratory factor analysis (EFA). Accordingly, we modified the OHIP-14 into the MOHIP-14^PW^. Finally, we compared their correlations with patient-reported oral health scores. *Results*: All OHIP-14 items had severely skewed distributions, and two had a correlation with the patient-reported oral health < 0.1. All seven pairs of items correlated well (0.47 to 0.62), but the Cronbach’s alphas indicated suboptimal reliability, with two below 0.70. CFA results offered suboptimal support to the original structure, and EFA found a three-dimensional structure best fitted the data. Therefore, we modified the OHIP-14 into the MOHIP-14^PW^. CFA on the MOHIP-14^PW^ offered stronger supports, and the Cronbach’s alphas increased to 0.92, 0.72, and 0.71. The MOHIP-14^PW^’s dimensions were more meaningful to pregnant women and had stronger relationships with patient-reported oral health than the OHIP-14; the average correlation coefficients increased by 26% from 0.19 in OHIP-14 to 0.24 in the MOHIP-14^PW^. *Conclusions*: The original OHIP-14 required modifications at the item-, dimension-, and measure- level, and the MOHIP-14^PW^ had better psychometric properties, easier interpretation, and stronger correlation with patient-reported oral health in low-income pregnant women. Through an interdisciplinary RWS on a large sample of pregnant women, this study offers concrete empirical evidence for the advantages of the MOHIP-14^PW^ over the original OHIP-14.

## 1. Introduction

Globally, oral diseases are highly prevalent and greatly reduce oral health-related quality of life (OHQoL) [1]. This relationship is particularly true for pregnant women, when their bodies undergo significant hormonal and physiological changes, affecting gingival inflammation [2,3]. The presence of dental and gingival disease negatively affects the self-perception of OHQoL in pregnant women, affecting their mental state and ability to achieve proper nutrition [2]. Low-income pregnant women are at particularly high risk of oral diseases and poor OHQoL [4].

While there are multiple OHQoL instruments [5,6,7,8,9], the Oral Health Impact Profile (OHIP) [10,11] is most widely used for both adults and pregnant women. According to Google Scholar, the original development article of the 49-item OHIP [10] and its shorter version, OHIP-14 [11], have been cited 2,873 and 2,590 times as of December 10, 2021, both with an average of approximately 100 citations per year. A newly published systematic review of adult OHQoL instruments found that the OHIP-14 was the most frequently used (26.8%), while the OHIP-49 ranked third [12]. In addition, the literature suggests that the “OHIP-14 appeared to be responsive to change” [13]. A recent systematic review of OHQoL during pregnancy included eight studies [2] and identified four OHQoL measures that have been used, with the OHIP-14 being the most common (62.5%). Additionally, some randomized controlled trials (RCTs) among pregnant women have used the OHIP-14 scores as the primary outcome [14].

However, we are uncertain if our tools for assessing OHQoL are accurate, both in the general population and in pregnant women. Despite its popularity, studies evaluating the psychometric properties of OHIP are rare [15,16]. Currently, it appears that there is no consensus regarding the dimensionality or the factor structure of the OHIP-14, i.e., which items belong to which dimensions [17]. For example, Santos et al. demonstrated the unidimensionality of the OHIP-14 in a Brazilian sample [18]. Brennan et al. found a multidimensional structure different from the original seven dimensions among a sample of South Australian dental patients [19]. In China, Xin and Ling identified a four-dimensional factor structure [20]. In addition, in another project [21] using the OHIP-14 in patients with severe periodontitis, the two corresponding authors (C.Y. and S.R.) and their team also found that the OHIP-14 had a factor structure other than the seven-dimensional structure proposed by the original author [11]. From a clinical aspect, the OHIP-14 offers seven dimension-scores and one total score, which is burdensome for busy clinicians and researchers. This burden can be eased in practical application if a simpler factor structure of the OHIP-14 can be identified for pregnant women resulting in fewer dimension-scores.

In this real-world study using empirical clinical data collected from two dental clinics, we aim to (1) thoroughly investigate the psychometric properties of the OHIP-14 in pregnant women at the item-, dimension-, and measure-level; (2) modify the scale accordingly into the MOHIP-14^PW^ (modified OHIP-14 for pregnant women); and (3) assess the relationship of patient-reported oral health with the OHIP-14 in comparison with the MOHIP-14^PW^ in low-income pregnant women. Finally, based on our findings, we propose further modifications of the MOHIP-14^PW^ for studies examining OHQoL among pregnant women.

## 2. Methods

### 2.1. Participant Recruitment

We recruited low-income pregnant women aged 18 years and above from two prenatal clinics on Long Island, New York. These clinics, located in suburban Nassau County on Long Island, serve Medicaid-eligible women. The Center for Maternal Oral Health (CMOH), a program within the dental practice to which women are referred, is in Great Neck, NY. This study received approval from Institutional Review Boards (IRB) at the New York University Medical Center and Long Island Jewish, North Shore, NY.

### 2.2. Data Collection

#### 2.2.1. Survey

We collected survey data via a written questionnaire provided to pregnant women as they visited the clinic for their prenatal care. We derived this questionnaire from those used previously [22]. Surveys were conducted in English and Spanish, and participants received a USD 5 gift card for participating and completing the survey. Aside from the Hispanic participants, all other pregnant women completed the survey in English. We included demographics in the questionnaire, such as age, race/ethnicity, place of birth, education, household income, marital status, and parity, i.e., number of live births. We also asked participants to complete the OHIP-14. In addition, all participants self-reported their overall condition of oral health by rating it into five categories: 1 = “excellent”, 2 = “very good”, 3 = “good”, 4 = “fair”, 5 = “poor”.

#### 2.2.2. Data Management

We transferred all data from the paper survey into an electronic dataset using the SPSS statistical software, version 25 [23]. To verify and clean all data, we used double-entry and comparisons.

### 2.3. Data Analysis

#### 2.3.1. Item-Level Analysis of the OHIP-14 and the Patient-Reported Oral Health

For the OHIP-14 and patient-reported oral health data, we calculated means, standard deviations (SD), medians, and distribution of responses (%) for each item. We considered an item as “good” if (1) responses were distributed well across the different categories without a severe floor or ceiling effect, i.e., less than 15% of participants choosing either the lowest or the highest response, and (2) the mean or median were close to the center of the range of response values [24]. For example, the OHIP-14 items should have a mean or median that is close to 2, because these items have response values as 0, 1, 2, 3, and 4.

#### 2.3.2. Dimension-Level Analysis of the OHIP-14 and the Patient-Reported Oral Health

We investigated the correlation matrix of the 14 OHIP items and patient-reported oral health, as well as the internal consistency of the seven dimensions using Cronbach’s alpha [25,26]. Prior literature indicates that a correlation coefficient of less than 0.1 is deemed small, between 0.1 and 0.3 medium, between 0.3 and 0.5 large [27]. Moreover, items measuring the same construct (dimension) should be more correlated than items outside the construct. Prior literature indicates that a Cronbach’s alpha between items measuring the same construct of greater than 0.7, 0.8, and 0.9 is deemed, respectively, as adequate, good, or excellent [27].

#### 2.3.3. Measure-Level Analysis of the OHIP-14

We investigated the factor structure of the OHIP-14 items by using confirmatory factor analysis (CFA) and exploratory factor analysis (EFA) techniques. Specifically, we used a CFA-EFA-ReCFA procedure [28], starting from a CFA according to the original factor structure of the OHIP-14 [11]. If the CFA results were not optimal, we ran an EFA to explore the potential factor structure that best fit this sample. If a new factor structure was found to better fit our sample, we then used an additional CFA (i.e., ReCFA) to confirm the newly identified factor structure. The following model fit index was used in the CFA-EFA-ReCFA procedure: normalized chi-square (NC) < 3.0, root mean square error of approximation (RMSEA) < 0.05, comparative fit index (CFI) > 0.95, Tucker–Lewis index (TLI) > 0.95, and standardized root mean square residual (SRMR) < 0.10 [27,29].

#### 2.3.4. Comparisons of the Relationship of Patient-Reported Oral Health with Dimension Scores from OHIP-14 versus MOHIP-14^PW^

After identification of the best factor structure for the data, we then calculated dimension scores using the mean of the items scores within each dimension. The correlation of the dimension scores with patient-reported oral health was then calculated and compared between the OHIP-14 and the MOHIP-14^PW^.

We used the *Mplus* software, Version 8 [30], for CFA and EFA, and the SPSS software, Version 25.0 [23], for all other statistical analysis.

## 3. Results

### 3.1. Demographics of Participants

In total, 291 pregnant women across two clinics participated in this study. We summarized their demographic properties in Table 1. On average, they were in their mid-twenties (26.5 ± 5.4 years old) and self-reported their race/ethnicity as Hispanic (42.3%), Black (32.1%), Asian (14.0%), and White (11.7%). Roughly half (53.1%) were U.S. immigrants. Most women had completed high school or above (82.1%), but more than two-thirds had a household income below USD 40,000/year. Over three-quarters were married or living as married, and roughly half (51.4%) did not have any previous live births.

### 3.2. Psychometric Properties of the OHIP-14 and the MOHIP-14^PW^

#### 3.2.1. Item Level

Table 2 displays the distribution of responses to the OHIP-14 items and the patient-reported oral health. All items of the OHIP-14 had severely skewed distributions among the five response categories (from 0 = “never” to 4 = “very often”), with a median of 0.0. The patient-reported oral health item showed excellent variability, with only one of the five response categories having either less than 5% or greater than 95% of participants, and with a median of 3.0 and mean of 2.94, just at the very center of the five categories (1, 2, 3, 4, 5).

#### 3.2.2. Dimension Level

Table 3 summarizes the Cronbach’s alpha, Spearman correlation matrix of the OHIP-14 items, and patient-reported oral health. All pairs of items within each of the original seven dimensions correlated well, with Spearman correlation coefficients ranging from 0.47 to 0.62. Cronbach’s alphas ranged from 0.64 to 0.77, with two dimensions (functional limitation and psychological disability) below 0.70, indicating suboptimal reliability. Two items (2 and 14) had Spearman correlation with the patient-reported oral health less than 0.1 (“small”).

#### 3.2.3. Measure-Level

Table 4 shows the factor analysis results. CFA results offered suboptimal support to the original conceptual seven-dimensional factor structure of the OHIP-14 in this sample: NC = 2.06, RMSEA = 0.061 (95% CI: 0.045, 0.077), CFI = 0.990, TLI = 0.984, and SRMR = 0.040. The RMSEA was greater than 0.05, which indicated the measurement model could be further optimized. Results from the EFA (data not shown, but available by request) indicated that at most four factors could be extracted from the 14 items and that a three-dimensional factor structure was the best fit for the data, with a 9-3-2 item allocation on the three new factors (Table 4).

Following this new three-dimensional factor structure, we modified the OHIP-14 into the modified OHIP-14 for pregnant women (MOHIP-14^PW^). We then ran another CFA for the MOHIP-14^PW^ and confirmed this new three-dimensional factor structure with stronger supports: NC = 1.56, RMSEA = 0.044 (95% CI: 0.028, 0.059), CFI = 0.993, TLI = 0.992, SRMR = 0.039. Each and all the model-fit indexes showed improvement, and the RMSEA in particular was below the cut-off value of 0.05. In addition, the three new dimensions in the MOHIP-14^PW^ had Cronbach’s alphas of 0.92, 0.72, and 0.71, indicating good to excellent reliability. We renamed the three new dimensions of the MOHIP-14^PW^ as “Physical Impact” (nine items), “Psychological Impact” (three items), and “Pain Impact” (two items).

### 3.3. Relationships of the Dimension Scores with Patient-Reported Oral Health

Table 5 displays the correlation of the MOHIP-14^PW^ with patient-reported oral health, compared to the correlation of the original OHIP-14 with patient-reported oral health. The MOHIP-14^PW^ was more strongly related to patient-reported oral health than the OHIP-14. The average correlation coefficients increased from 0.19 (range: 0.13–0.28) in OHIP-14 to 0.24 (range: 0.21–0.29) in MOHIP-14^PW^, a 26% improvement.

## 4. Discussion

In this real-world study, we found that the original factor structure of the OHIP-14 was not optimally suited for this surveyed sample of low-income pregnant women. We therefore modified the OHIP-14 into the MOHIP-14^PW^, which has a three-dimensional factor structure that was more suitable for the underlying data. This led to changes of the OHIP-14 scores in the following ways: (1) number of subscales, from seven to three; (2) re-grouping of the items included in OHIP-14; and (3) range of the subscale scores, from 0–8 for all the original seven subscales to 0–8, 0–12, and 0–36 for the three new subscales.

Scale adaptation and modification is a cutting-edge science [31], and it is common practice in scale development [24,32]. A good example is the modified dental anxiety scale (MDAS) [33], which was modified from the original DAS [34,35]. Moreover, modification of a scale can be critical at resecuring positive findings, such as a large randomized controlled trial (RCT) on Parkinson’s disease [36].

### 4.1. Interpretation of the New Three-Dimensional Factor Structure of the MOHIP-14^PW^

From a content perspective, the primary change from the OHIP-14 to the MOHIP-14^PW^ is how items are grouped into domains. Specifically, both items 9 and 10 (“difficulty in relaxing due to dental problems” and “embarrassment due to dental issues”) were originally part of the Psychological Disability domain in the OHIP-14. In the MOHIP-14^PW^, item 9 was included instead in the Physical Impact domain and item 10 into Psychological Impact instead of Psychological Disability.

From a technical and psychometrical perspective, the separation of items 9 and 10 is not surprising if we have a closer look at the item matrix (Table 3). Item 9 is more correlated with items from other dimensions than Item 10. Similarly, Item 10 is more correlated with Items 5 (0.55) and 6 (0.54) than Item 9 (0.47). These correlation patterns are precisely captured in the new dimension structure of the MOHIP-14^PW^. As a result, both Items 9 and 10 had higher factor loadings in their new dimensions of the MOHIP-14^PW^ compared to their factor loadings in the OHIP-14 (Table 4)—the factor loading on Item 9 increased from 0.870 to 0.906, while the factor loading on Item 10 increased from 0.761 to 0.947. All these technical outputs offered empirical supports for the new dimension structure of the MOHIP-14^PW^.

A key question is whether Item 9 (“difficulty in relaxing due to dental problems”) conceptually is a psychological or physical attribute. From the content of Item 9, there can be an argument made either way. Hence, which domain Item 9 is grouped into likely depends strongly on the underlying empirical data. The data here suggest that at least for our sample of low-income pregnant women, difficulty in relaxing due to dental problems is primarily a physical rather than psychological issue.

### 4.2. Advantages and Innovations of the MOHIP-14^PW^

Several studies have used the OHIP-14 to investigate the OHQoL among pregnant women [2]. As evidenced in our study, the MOHIP-14^PW^ has the following advantages and innovations over the original OHIP-14 among the pregnant women: (1) improved psychometric properties; (2) easier interpretation for pregnant women; and (3) increased relationship with patient-reported oral health. We hope the field will benefit in the future from these important innovations and advantages of the MOHIP-14^PW^.

### 4.3. Future Directions

Obtaining data from several hundred pregnant women (*n* = 291) participating in this study is a meaningful achievement. We are fortunate and uniquely advantaged by having access to large numbers of pregnant women seeking dental care in a metropolitan area. In the future, we plan to collect surveys from other patient groups.

We suggest that modifying the response to the 14 items, from the 5-point Likert type (0, 1, 2, 3, 4) to a continuous scale, such as 0–100, where 0 = “never”, and 100 = “all the time”, would improve the MOHIP-14^PW^ further. The 14 items in the original OHIP-14 all exhibited severely skewed response distributions with a median of 0.0 in this population, which limits the ability of future studies to detect any changes in oral health-related quality of life. However, this severe skewness issue would be easily rectified by modifying the responses to all 14 items from a five-point Likert scale to a continuous scale, such as 0 to 100.

Such a modification to the response scale has been used previously by the lead and corresponding author (C.Y.), which was proven to be critical in avoiding the very likely failure of a large RCT [37]. In that RCT, although with positive findings (i.e., *p* < 0.05) on the primary outcome measure, the improvements are all from above 90 out of the 100-point scale, such as from 90.2 to 92.1. Had the original primary measure, the Nolan self-efficacy scale [38], not been modified from the original five-point (1, 2, 3, 4, 5) Likert-scale to the 100-point continuous scale [37], the large RCT would not have been able to detect any changes in the primary outcome. As Kline (2015) ([27], p. 91) illustrated, reliability of a dimension in a measure, as assessed by Cronbach’s alpha, is a function of two parameters: (1) number of items within the dimension; and (2) average Pearson correlation between all pairs of items within the dimension. By definition, the five-point Likert responses are categorical, not continuous. Thus, Pearson correlation and Cronbach’s alpha are not as appropriate for a five-point Likert scale. Instead, a 0–100 continuous scale is much more appropriate for Pearson correlation and Cronbach’s alpha. Hence, we consider that future work examining changes in OHQoL should consider further modifying the MOHIP-14^PW^ to allow for a continuous response scale.

We plan to eliminate the items that showed low correlation with the patient-reported oral health, especially items 2 and 14. Close correlation with the content measure (patient-reported oral health) is the fundamental criteria for the inclusion or exclusion of an item. In a similar study [39] on the National Institutes of Health Stroke Scale (NIHSS [40]), 1 of the 15 items was negatively correlated with all other items. After that item was eliminated, the patient-reported performance of the measure improved substantially. Similarly, in another study of the Repeatable Battery for the Assessment of Neuropsychological Status (RBANS [41]) in Parkinson’s disease, half of the 12 items were eliminated due to low correlation with other items. The resulting product exhibited much better performance than the original RBANS [28].

Many factors are related to oral health, including but not limited to age, race/ethnicity, education, diet, and income. Specifically, oral health in low-income pregnant women might be significantly different from that seen in higher-income pregnant women. Moreover, other factors may be associated with more changes to oral health in low-income pregnant women compared to higher-income pregnant women. Therefore, our team has started another study to investigate these potential relationships. Since the original OHIP-14 appeared to be responsive to change [13], we believe that the MOHIP-14^PW^ will maintain this important feature. In the future, we also plan to add a new survey to assess these pregnant women’s perceptions of changes in their oral health following treatment at the clinics, so that we can evaluate the response of MOHIP-14^PW^ to change. In addition, future work examining the MOHIP-14^PW^ for pregnant women should consider avoiding the result deviation caused by language problems across different populations, cultures, and countries by offering the tool in more languages.

We also plan to apply the further-refined MOHIP-14^PW^ onto national and international samples of pregnant women. Broader sampling across sociodemographic backgrounds and geography is warranted to further refine the MOHIP-14^PW^ for this population. Since the women recruited in this sample may only be representative of low-income pregnant women in suburban New York, future work examining the MOHIP-14^PW^ for pregnant women should consider recruiting nationally and internationally representative populations. Such data would enable researchers to examine differential item functioning (i.e., measurement bias at the item level) across different populations, cultures, and countries.

## 5. Conclusions

In a real-world study with empirical data from a relatively large sample, we modified the popular OHIP-14 into the MOHIP-14^PW^ through full-scale psychometric assessment and refinement. This modification improved both the psychometric properties and relationship with patient-reported oral health for a sample of low-income pregnant patients. This new MOHIP-14^PW^ should be considered for use in future studies in pregnant women for more accurate and meaningful measure of OHQoL, especially if further tested in more diverse populations. The clinical aspects of this research include a decreased burden through the reduction of dimension-scores from seven to three, as well as easier understanding and easier interpretation of the dimensions for pregnant women. The purpose of collecting the MOHIP-14^PW^ is to investigate OHQoL among pregnant women. This scale can be used either in clinical practice or research to ease the burden for clinicians, researchers, and pregnant women.

## Figures and Tables

**Table 1:** Demographic characteristics of the study participants (N = 291).

Demographic Characteristics	Mean ± SD/*n* (%)
Age (years)	26.5 ± 5.4
Race/ethnicity	
White	31 (11.7%)
Black	85 (32.1%)
Asian	37 (14.0%)
Hispanic	112 (42.3%)
Place of birth	
USA	136 (46.9%)
Puerto Rico (USA)	9 (3.1%)
Caribbean (other than Puerto Rico)	40 (13.8%)
Central/South America	59 (20.3%)
South Asia	22 (7.6%)
Other Asia	16 (5.5%)
Other	8 (2.8%)
Education	
Lower than high school	52 (17.9%)
High school/some college	182 (62.8%)
College graduate or above	56 (19.3%)
Household income	
≤USD 15,000/year	88 (30.2%)
USD 15,001–40,000/year	109 (37.5%)
≥USD 40,001/year	94 (32.3%)
Marital status	
Married/living as married	225 (77.6%)
Others	65 (22.4%)
Parity	
No previous live births	148 (51.4%)
1 previous live birth	73 (25.3%)
≥2 previous live births	67 (23.3%)

**Table 2:** Distribution of responses to the OHIP-14 items among the study participants.

OHIP-14 Item	Brief Description	N	Mean	SD	Median	Distribution of Responses (%)				
						0	1	2	3	4
1	Difficulty in pronunciation	283	0.16	0.48	0.0	88.0	8.8	2.5	0.7	0.0
2	Deterioration of taste	278	0.23	0.62	0.0	85.3	9.0	4.0	1.4	0.4
3	Pain in the mouth	277	0.56	0.89	0.0	66.8	14.1	16.2	2.2	0.7
4	Discomfort while chewing food	277	0.43	0.86	0.0	76.2	9.7	10.8	1.8	1.4
5	Self-conscious about teeth, mouth, and dentures	275	0.63	1.09	0.0	68.7	11.3	13.1	2.5	4.4
6	Tensed due to problems of your teeth and mouth	277	0.38	0.82	0.0	77.3	12.3	6.9	2.2	1.4
7	Diet unsatisfactory due to dental problems	277	0.18	0.58	0.0	88.1	7.2	3.6	0.4	0.7
8	Interruption during meals due to dental problems	276	0.33	0.77	0.0	81.2	8.7	8.0	0.7	1.4
9	Difficulty in relaxing due to dental problems	277	0.22	0.56	0.0	84.5	10.5	4.0	1.1	0.0
10	Embarrassment due to dental issues	278	0.42	0.85	0.0	74.8	12.9	9.0	1.4	1.8
11	Irritability with others due to dental problems	277	0.18	0.57	0.0	88.4	6.5	3.2	1.8	0.0
12	Difficulty in doing usual jobs due to dental problems	278	0.13	0.46	0.0	91.4	5.8	1.8	1.1	0.0
13	Less satisfaction in life due to dental issues	278	0.17	0.53	0.0	88.8	6.8	3.6	0.4	0.4
14	Total unable to function due to dental issues	278	0.12	0.44	0.0	91.7	4.7	3.2	0.4	0.0
Patient-reported oral health	Overall condition of your oral health					1	2	3	4	5
		290	2.94	0.90	3.00	6.9	20.0	47.9	22.4	2.8

Likert-type response coding (Slade, 1997): 0 = “never”, 1 = “hardly ever”, 2 = “occasionally’, 3 = “fairly often”, and 4 = “very often”. Overall condition of your oral health: 1 = “excellent”, 2 = “very good”, 3 = “good”, 4 = “fair”, 5 = “poor”.

**Table 3:** OHIP-14 in the study participants: Cronbach’s alpha of the original 7 dimensions, and Spearman correlation matrix of the 14 items and the overall health.

OHIP-14 Item	1	2	3	4	5	6	7	8	9	10	11	12	13	14
1		(0.67)												
2	**0.50**													
3	0.28	0.34		(0.72)										
4	0.36	0.38	**0.56**											
5	0.34	0.40	0.22	0.44		(0.70)								
6	0.40	0.45	0.32	0.53	**0.54**									
7	0.45	*0.52*	0.29	0.46	0.39	0.50		(0.72)						
8	0.46	*0.50*	0.39	*0.58*	0.40	0.45	**0.56**							
9	*0.58*	*0.56*	0.41	*0.51*	0.35	*0.51*	*0.51*	*0.63*		(0.64)				
10	0.27	0.34	0.31	*0.51*	*0.55*	*0.54*	0.40	0.38	**0.47**					
11	0.47	*0.52*	0.26	0.47	0.44	*0.56*	0.55	*0.61*	*0.59*	*0.49*		(0.74)		
12	*0.57*	0.48	0.33	0.45	0.30	0.42	*0.63*	0.51	*0.53*	0.40	**0.59**			
13	0.47	0.45	0.27	0.40	0.36	0.45	*0.64*	0.49	*0.57*	*0.53*	0.58	*0.64*		(0.77)
14	*0.50*	*0.52*	0.34	0.43	0.32	0.43	*0.61*	0.49	*0.54*	0.44	*0.61*	*0.67*	**0.62**	
Overall oral health	0.14	0.07	0.22	0.15	0.21	0.21	0.12	0.16	0.11	0.28	0.14	0.17	0.18	0.09

Values in bold are Spearman correlation coefficients within the same original OHIP-14 dimensions. Values in *italic* indicate Spearman correlation coefficients higher than those within the same original OHIP-14 dimensions. Values in parentheses are the Cronbach alphas for each of the seven original OHIP-14 dimensions.

**Table 4:** Factor structures of the OHIP-14 in pregnant women: original vs. modified.

Item #	Original Dimension		Modified Dimension	
	Dimension Name	Factor Loading	Dimension Name	Factor Loading
1	Functional Limitation	0.834	Physical Impact	0.804
2	Functional Limitation	0.867	Physical Impact	0.834
3	Physical Pain	0.723	Pain Impact	0.720
4	Physical Pain	1.004	Pain Impact	1.008
5	Psychological Discomfort	0.734	Psychological Impact	0.724
6	Psychological Discomfort	0.895	Psychological Impact	0.889
7	Physical Disability	0.859	Physical Impact	0.854
8	Physical Disability	0.900	Physical Impact	0.897
9	Psychological Disability	0.870	Physical Impact	0.906
10	Psychological Disability	0.761	Psychological Impact	0.853
11	Social Disability	0.922	Physical Impact	0.924
12	Social Disability	0.961	Physical Impact	0.963
13	Handicap	0.904	Physical Impact	0.889
14	Handicap	0.961	Physical Impact	0.947
Model Fit Indexes				
	Chi-Square	115.364		115.276
	Df	56		74
	NC	2.060		1.558
	RMSEA (95% CI)	0.061 (0.045, 0.077)		0.044 (0.028, 0.059)
	CFI	0.990		0.993
	TLI	0.984		0.992
	SRMR	0.040		0.039

NC: normalized chi-square; RMSEA: root mean square error of approximation; CI: confidence interval; CFI: comparative fit index; TLI: Tucker–Lewis index; SRMR: standardized root mean square residual.

**Table 5:** Comparisons of the relationship of overall oral health with dimension scores from OHIP-14 versus MOHIP-14 in pregnant women (MOHIP-14^PW^).

Original: OHIP-14		Modified: MOHIP-14^PW^	
Dimension Name	Spearman Correlation with Overall Oral Health	Dimension Name	Spearman Correlation with Overall Oral Health
Functional Limitation	0.13	Physical Impact	0.23
Physical Pain	0.21	Psychological Impact	0.29
Psychological Discomfort	0.23	Pain Impact	0.21
Physical Disability	0.16		
Psychological Disability	0.28		
Social Disability	0.16		
Handicap	0.19		
Average	0.19	Average	0.24
Range	(0.13, 0.28)	Range	(0.21, 0.29)

## Data Availability

The data presented in this study are available on request from the corresponding authors. The data are not publicly available due to other ongoing investigations and manuscripts.

## References

[1] Peres M.A., Macpherson L.M., Weyant R.J., Daly B., Venturelli R., Mathur M.R., Listl S., Celeste R.K., Guarnizo-Herreño C.C., Kearns C. (2019). Oral diseases: A global public health challenge. Lancet.

[2] Fakheran O., Saied-Moallemi Z., Khademi A., Sahebkar A. (2020). Oral Health-Related Quality of Life during Pregnancy: A Systematic Review. Curr. Pharm. Des..

[3] Acharya S., Bhat P.V. (2009). Oral-health-related quality of life during pregnancy. J. Public Health Dent..

[4] Russell S.L., Kerpen S.J., Rabin J.M., Burakoff R.P., Yang C., Huang S.S. (2021). A Successful Dental Care Referral Program for Low-Income Pregnant Women in New York. Int. J. Environ. Res. Public Health.

[5] Slade G.D., Strauss R.P., Atchison K., Kressin N., Locker D., Reisine S.T. (1998). Conference summary: Assessing oral health outcomes--measuring health status and quality of life. Community Dent. Health.

[6] Thomson W.M., Broder H.L. (2018). Oral–Health–Related Quality of Life in children and adolescents. Pediatric Clin..

[7] Yang C., Crystal Y., Ruff R., Veitz-Keenan A., McGowan R., Niederman R. (2020). Quality Appraisal of Child Oral Health–Related Quality of Life Measures: A Scoping Review. JDR Clin. Transl. Res..

[8] Haag D., Peres K., Balasubramanian M., Brennan D. (2017). Oral Conditions and Health-Related Quality of Life: A Systematic Review. J. Dent. Res..

[9] Tsakos G., Allen F., Peres M.A., Watt R.G. (2021). Oral Health-Related Quality of Life, in Oral Epidemiology.

[10] Slade G.D., Spencer A.J. (1994). Development and evaluation of the oral health impact profile. Community Dent. Health.

[11] Slade G.D. (1997). Derivation and validation of a short-form oral health impact profile. Community Dent. Oral Epidemiol..

[12] Riva F., Seoane M., Reichenheim M.E., Tsakos G., Celeste R.K. (2021). Adult oral health-related quality of life instruments: A systematic review. Community Dent. Oral Epidemiol..

[13] Locker D., Jokovic A., Clarke M. (2004). Assessing the responsiveness of measures of oral health-related quality of life. Community Dent. Oral Epidemiol..

[14] Musskopf M.L., Milanesi F.C., da Rocha J.M., Fiorini T., Moreira C.H.C., Susin C., Rösing C.K., Weidlich P., Oppermann R.V. (2018). Oral health related quality of life among pregnant women: A randomized controlled trial. Braz. Oral Res..

[15] Schierz O., Baba K., Fueki K. (2021). Functional Oral Health-Related Quality of Life Impact: A Systematic Review in Populations with Tooth Loss. J. Oral Rehabil..

[16] John M.T., Reißmann D.R., Feuerstahler L., Waller N., Baba K., Larsson P., Čelebić A., Szabo G., Rener-Sitar K. (2014). Factor analyses of the Oral Health Impact Profile–Overview and studied population. J. Prosthodont. Res..

[17] Soares G., Santiago P., Werneck R., Michel-Crosato E., Jamieson L. (2021). A psychometric network analysis of OHIP-14 across Australian and Brazilian populations. JDR Clin. Transl. Res..

[18] Santos C.M.d., de Oliveira B.H., Nadanovsky P., Hilgert J.B., Celeste R.K., Hugo F.N. (2013). Oral Health Impact Profile-14::¿ una escala unidimensional?. Cad. D Saúde. Pública..

[19] Brennan D.S., Spencer A.J. (2004). Dimensions of oral health related quality of life measured by EQ-5D+ and OHIP-14. Health Qual. Life Outcomes.

[20] Xin W., Ling J. (2006). Validation of a Chinese version of the oral health impact profile-14. Chin. J. Stomatol..

[21] Sousa F., Yang C., Oliveria V.B., Russell S.L., Niederman R., Rego R.O. Severe Periodontitis and OHRQoL Measured by the OHIP-14. Proceedings of the 2020 IADR/AADR/CADR General Session.

[22] Boggess K.A., Urlaub D.M., Massey K.E., Moos M.-K., Matheson M.B., Lorenz C. (2010). Oral hygiene practices and dental service utilization among pregnant women. J. Am. Dent. Assoc..

[23] IBM (2017). IBM SPSS Statistics for Window.

[24] DeVellis R.F., Thorpe C.T. (2021). Scale Development: Theory and Applications.

[25] Cronbach L.J. (1951). Coefficient alpha and the internal structure of tests. Psychometrika.

[26] Cronbach L.J., Shavelson R.J. (2004). My current thoughts on coefficient alpha and successor procedures. Educ. Psychol. Meas..

[27] Kline R.B. (2015). Principles and Practice of Structural Equation Modeling.

[28] Yang C., Garrett-Mayer E., Schneider J.S., Gollomp S.M., Tilley B.C. (2009). Repeatable battery for assessment of neuropsychological status in early Parkinson’s disease. Mov. Disord..

[29] Brown T.A. (2015). Confirmatory Factor Analysis for Applied Research.

[30] Muthén L.K., Muthén B.O. (2017). Mplus User’s Guide (1998–2017).

[31] Stewart A.L., Thrasher A.D., Goldberg J., Shea J.A. (2012). A framework for understanding modifications to measures for diverse populations. J. Aging Health.

[32] Yang C., Ford M.E., Tilley B.C., Greene R.L. (2016). Religiosity in black and white older Americans: Measure adaptation, psychometric validation, and racial difference. Medicine.

[33] Humphris G.M., Morrison T., Lindsay S. (1995). The Modified Dental Anxiety Scale: Validation and United Kingdom norms. Community Dent. Health.

[34] Corah N.L., Gale E.N., Illig S.J. (1978). Assessment of a dental anxiety scale. J. Am. Dent. Assoc..

[35] Corah N.L. (1969). Development of a dental anxiety scale. J. Dent. Res..

[36] Yang C., Vrana K.E. (2018). Rescuing Suboptimal Patient-Reported Outcome Instrument Data in Clinical Trials: A New Strategy. Healthcare.

[37] Green M.J., van Scoy L.J., Foy A.J., Stewart R.R., Sampath R., Schubart J.R., Lehman E.B., Dimmock A.E.F., Bucher A.M., Lehmann L.S. (2018). A randomized controlled trial of strategies to improve family members’ preparedness for surrogate decision-making. Am. J. Hosp. Palliat. Med..

[38] Nolan M.T., Hughes M.T., Kub J., Terry P.B., Astrow A., Thompson R.E., Clawson L., Texeira K., Sulmasy D.P. (2009). Development and validation of the family decision-making self-efficacy scale. Palliat. Supportive Care.

[39] Yang C., Zhao W. On the relationship between the modified Rankin scale (mRS) and the NIH stroke scale (NIHSS). Proceedings of the 36th Annual Meeting of the Society for Clinical Trials (SCT), The Society for Clinical Trials (SCT).

[40] Brott T., Adams H.P., Olinger C.P., Marler J.R., Barsan W.G., Biller J., Spilker J., Holleran R., Eberle R., Hertzberg V. (1989). Measurements of acute cerebral infarction: A clinical examination scale. Stroke.

[41] Randolph C., Tierney M.C., Mohr E., Chase T.N. (1998). The Repeatable Battery for the Assessment of Neuropsychological Status (RBANS): Preliminary clinical validity. J. Clin. Exp. Neuropsychol..

